# Large root cortical cells and reduced cortical cell files improve growth under suboptimal nitrogen in silico

**DOI:** 10.1093/plphys/kiad214

**Published:** 2023-04-11

**Authors:** Ivan Lopez-Valdivia, Xiyu Yang, Jonathan P Lynch

**Affiliations:** Department of Plant Science, The Pennsylvania State University, University Park, PA 16802, USA; Department of Plant Science, The Pennsylvania State University, University Park, PA 16802, USA; Department of Plant Science, The Pennsylvania State University, University Park, PA 16802, USA

## Abstract

Suboptimal nitrogen availability is a primary constraint to plant growth. We used *OpenSimRoot*, a functional-structural plant/soil model, to test the hypothesis that larger root cortical cell size (CCS), reduced cortical cell file number (CCFN), and their interactions with root cortical aerenchyma (RCA) and lateral root branching density (LRBD) are useful adaptations to suboptimal soil nitrogen availability in maize (*Zea mays*). Reduced CCFN increased shoot dry weight over 80%. Reduced respiration, reduced nitrogen content, and reduced root diameter accounted for 23%, 20%, and 33% of increased shoot biomass, respectively. Large CCS increased shoot biomass by 24% compared with small CCS. When simulated independently, reduced respiration and reduced nutrient content increased the shoot biomass by 14% and 3%, respectively. However, increased root diameter resulting from large CCS decreased shoot biomass by 4% due to an increase in root metabolic cost. Under moderate N stress, integrated phenotypes with reduced CCFN, large CCS, and high RCA improved shoot biomass in silt loam and loamy sand soils. In contrast, integrated phenotypes composed of reduced CCFN, large CCS, and reduced LRBD had the greatest growth in silt loam, while phenotypes with reduced CCFN, large CCS, and high LRBD were the best performers in loamy sands. Our results support the hypothesis that larger CCS, reduced CCFN, and their interactions with RCA and LRBD could increase nitrogen acquisition by reducing root respiration and root nutrient demand. Phene synergisms may exist between CCS, CCFN, and LRBD. CCS and CCFN merit consideration for breeding cereal crops with improved nitrogen acquisition, which is critical for global food security.

## Teaser

Enlarged root cortical cells and reduced cortical cell file number decrease the metabolic cost of soil exploration, thereby improving plant growth under suboptimal nitrogen availability.

## Introduction

The development of crops with reduced fertilizer requirements is needed in global agriculture to reduce the environmental, economic, and energy costs of crop production in high-input agroecosystems and to increase crop production in low-input agroecosystems (Koevoets et al. 2016; Lynch 2019, 2022b). One avenue toward this goal is via the selection of root phenotypes that reduce the metabolic cost of soil exploration (Lynch 2015; Lynch et al. 2021, Lynch 2022a). The metabolic costs of root tissue can be estimated as the amount of resources, mainly carbohydrates and mineral nutrients, needed in root growth and maintenance (Chimungu, et al. 2015b; Lynch 2015; Postma and Lynch 2011a, 2011b; Saengwilai et al. 2014a; Zhu et al. 2010a). The carbon cost of soil exploration includes carbon expenditure in root tissue construction and maintenance and ion uptake and assimilation (Nielsen et al. 1994, 2001). Maintenance respiration is the largest carbon cost over time. Root metabolic costs at low nutrient availability are substantially greater than those at high nutrient availability (Nielsen et al. 2001; Lambers et al. 2008). Root respiration is also a major cause of growth reduction under nutrient stress (Postma and Lynch 2011a). The consumption of carbon by root respiration can exceed 50% of daily photosynthesis under suboptimal nutrient levels (Nielsen et al. 2001; Ho et al. 2004). Therefore, phenes are the basic unit of the phenotype and phene states are variants for those phenes (Lynch 2011; York et al. 2013; Rangarajan and Lynch 2021) that reduce maintenance respiration and allow better root growth and soil exploration (Pieruschka and Poorter 2012; York et al. 2013; Fonta et al. 2022a), thus improving crop growth under limited resource availability, and therefore present opportunities for the development of crops with reduced nutrient requirements (Lynch 2015, 2022b).

The “Steep, Cheap, and Deep” (SCD) ideotype proposes maize (*Zea mays*) root phenotypes to optimize water and N capture under limited availability of those resources (Lynch 2013). This ideotype consists of root anatomical, architectural, and physiological phenotypes that increase root depth, and improve the acquisition of resources from deep soil domains (Schäfer et al. 2022a). By determining the proportion of respiring to nonrespiring root tissue and nutrient cost of tissue construction and maintenance, root anatomy regulates the metabolic cost of soil exploration and therefore is an important factor for plant adaptation under edaphic stress (Fan et al. 2003; Mano et al. 2006; Jaramillo et al. 2013; Galindo-Castañeda et al. 2018; Lynch et al. 2021). The “topsoil foraging” ideotype for *P* capture (Lynch and Brown 2001; Ho et al. 2004; Wang et al. 2010; Lynch 2011, 2022b; Richardson et al. 2011; Lynch 2019) has been useful as a breeding goal in developing soybean and common bean cultivars that can enhance *P* acquisition in low phosphorus and drought environments (Burridge et al. 2019), with similar application for enhanced *P* acquisition in maize (Zhu et al. 2005; Sun et al. 2018; Lynch 2022b), given that *P* is immobile in the soil strata, and is concentrated in the topsoil. Phene states that improve root exploitation of the topsoil, such as shallow root angle (Lynch and Brown 2001; Rubio et al. 2003; Zhu et al. 2005; Rangarajan et al. 2018), many hypocotyl-borne roots (Miller et al. 2003; Walk et al. 2006; Rangarajan et al. 2018), dense lateral branching (Zhu and Lynch 2004; Jia et al. 2018; Mohammed et al. 2022; Schäfer et al. 2022b), greater production of axial roots (Walk et al. 2006; Miguel et al. 2015; Sun et al. 2018; Rangarajan et al. 2018; Mohammed et al. 2022; Schäfer et al. 2022b), RCA formation (Postma and Lynch 2011a, 2011b), and root hair formation (Zhu et al. 2010b; Miguel et al. 2015; Mohammed et al. 2022), have greater *P* capture. Anatomical phene states that contribute to reduced metabolic cost were also found to exhibit synergism with architectural phenes (Postma and Lynch 2011a).

The formation of root cortical aerenchyma (RCA), the enlarged intercellular spaces that form through either programmed cell death or cell separation (Evans 2004), is generally increased in response to hypoxia (Jackson and Armstrong 1999) and various edaphic stresses, including suboptimal availability of phosphorus, nitrogen, sulfur, and water (Konings and Verschuren 1980; Drew et al. 1989; Bouranis et al. 2003; Fan et al. 2003; Zhu et al. 2010a; Saengwilai et al. 2014a; Chimungu et al. 2015a; Galindo-Castañeda et al. 2019). RCA formation alleviates the limitation of hypoxia for root respiration with improved oxygen transport (Jackson and Armstrong 1999). The utility of RCA formation to maintain greater growth rates under various soil nutrient and drought stresses by remobilizing nutrients from the root cortex and reducing maintenance respiration has been demonstrated in several previous studies (Fan et al. 2003; Zhu et al. 2010a; Postma and Lynch 2011a; Jaramillo et al. 2013; Saengwilai et al. 2014a; Chimungu et al. 2015a; Galindo-Castañeda et al. 2019; Sidhu et al. 2023). However, the dynamic interaction between RCA and other anatomical phenes and their effects on growth requires further examination, as RCA formation reduces the proportion of root volume occupied by living cortical tissue, which is more metabolically demanding than stelar tissue (Lynch 2013). Chimungu et al. (2014a, 2014b) and Sidhu et al. (2023) reported that a reduction in the number of concentric layers of parenchyma cells in the cortex of the maize root, or cortical cell file number (CCFN), and increased volume of individual cortical parenchyma cells, or cortical cell size (CCS), could decrease the metabolic costs of root growth and maintenance, in terms of both the carbon cost of root respiration and the nutrient content of cortical tissue. Contrasting maize lines exposed to water deficit stress in controlled environments and the field, larger CCS, and reduced CCFN were associated with reduced root respiration, deeper rooting, greater water capture, improved plant water status, and hence greater growth and yield (Chimungu et al. 2014a, 2014b). However, the physiological utilities of larger CCS, reduced CCFN, and their interaction with RCA and root architectural phenes under nutrient deficiencies, are not known.

The utility of a root phene state under stress may be dependent on its interactions with other architectural and anatomical phenes (Rangarajan et al. 2018, 2022). Phene synergism refers to the phenomenon where the combined effect of 2 or more phenes is greater than the additive sum of their individual effects (Rangarajan et al. 2018; Ajmera et al. 2022; Schäfer et al. 2022a). For example, in low *P* soils, common bean genotypes with long root hairs (RHL) and shallow basal root growth angle (BRGA) had 3-fold greater biomass accumulation than genotypes with short root hairs and steep root angle, while only 89% greater biomass was contributed by RHL alone, and 58% by shallow BRGA alone (Miguel et al. 2015). In another study, RCA formation in lateral roots in genotypes with increased lateral root branching density (LRBD) had greater benefits for phosphorus acquisition (Postma and Lynch 2011b) than the effect of RCA alone. Integration of anatomical phenes and architectural phenes of maize root systems are important for nitrogen and water acquisition (York et al. 2013, 2016; Klein et al. 2020; Ajmera et al. 2022; Fonta et al. 2022b). These potential synergisms may be useful for breeding crops with greater edaphic stress tolerance (Lynch 2022b). However, interactions among phenes may also be antagonistic, i.e. the functional response of phene states in combination is worse than that expected from the sum of their responses in isolation. For example, at low soil N levels, a phenotype with increased LRBD combined with RCA formation caused 42% reduction in shoot dry weight, compared with the expected additive effects of this phenotype, which indicates a functional antagonism (Postma and Lynch 2011b; York et al. 2013). Therefore, understanding the interactions of CCFN and CCS with architectural phenes, such as LRBD and BRGA, is fundamental to finding ideotypes for future climates.

The integrated phenotype is the sum of all the phenes states that constitute a particular root system. The phene interactions with each other and with the environment will determine fitness (Lynch 2019). Since the combination of integrated phenotypes and environments constitute a very large number of scenarios, it is difficult to explore the fitness landscape in the greenhouse or field conditions. However, structural-functional models permit the evaluation of all possible combinations of integrated phenotypes and their performance in multiple environments (Rangarajan et al. 2022). *OpenSimRoot* (OSR) is a structural-functional plant model that simulates the spatial-temporal development of a root system growing in a particular soil and atmosphere (Postma et al. 2017). OSR has been used to explore the fitness landscape in several species such as common bean (*Phaseolus vulgaris*; Rangarajan et al. 2018, 2022; Rangarajan and Lynch 2021), rice (*Oryza sativa*; Ajmera et al. 2022), squash (*Cucurbita* spp.; Postma and Lynch 2012), barley (*Hordeum vulgare*; Schneider et al. 2017), *Arabidopsis thaliana* (Ma et al. 2001), and maize (Postma and Lynch 2011b; Postma et al. 2014; Rangarajan and Lynch 2021; Rangarajan et al. 2022; Schäfer et al. 2022a). OSR is a useful tool to evaluate the fitness landscape of phenotypes potentially useful as ideotypes for crop breeding.

A quantitative understanding of the functional dependence of 1 phenotype on the expression of other phenotypes, as well as interactions with environmental factors is important to understand the utility of phenotypic diversity for plant breeding. We hypothesize that reduced CCFN and larger CCS, in combination with RCA formation, would decrease root respiration and tissue nutrient content, which would result in greater root growth, more efficient acquisition of soil nitrogen, and better root and whole plant establishment under suboptimal nitrogen availability. OSR, a functional-structural plant model, was used to evaluate: (i) the elemental functions of CCFN and CCS, (ii) the utility of CCS, CCFN, and RCA under suboptimal nitrogen availability, and (iii) potential synergism between CCS, CCFN, and LRBD.

## Results

### Model to predict phene states of CCFN and CCS

**Figure 1.**
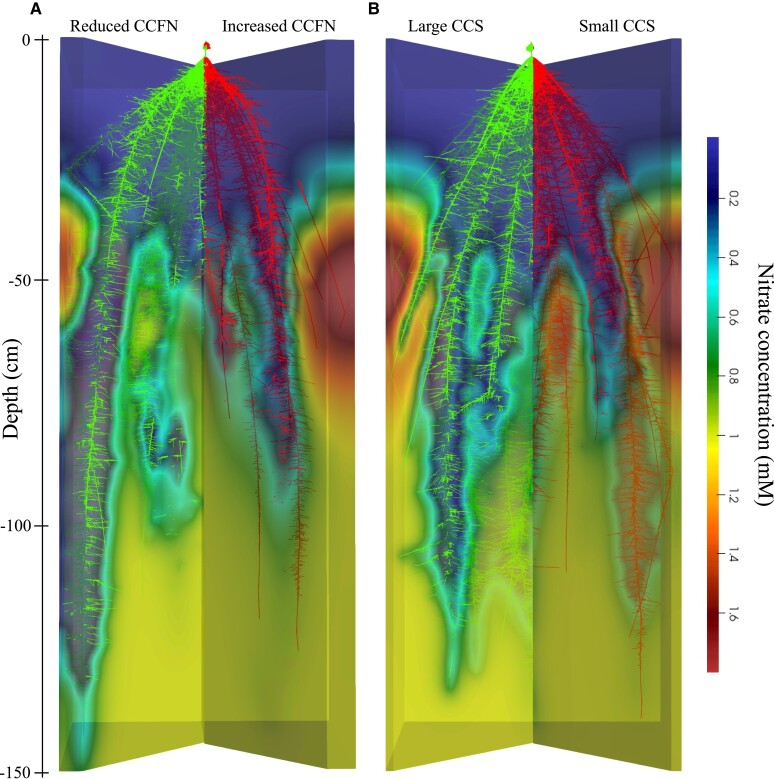
Interaction of root anatomical phenotypes, root growth, and soil nitrogen availability. The visualized output of the simulated root architecture of **A)** reduced CCFN, increased CCFN, **B)** Large CCS, and small CCS under moderate N stress. The continuous gradient in the simulated soil represents nitrogen depletion by root uptake and leaching.

OSR simulates the effect of anatomical phenotypes in the whole root system as the nutrient and carbon cost of tissue construction and maintenance. To simulate the phene states of CCFN and CCS (Fig. 1), we empirically measured respiration, nitrogen content, and root diameter in 12 maize genotypes with phenotypic variation for CCFN and CCS (Supplemental Fig. S1 and Table S1). Then, we built a multiple linear regression model to predict the tissue construction and maintenance cost for reduced/increased CCFN and small/large CCS (Table 1 and Supplemental Fig. S2). The model predicted that reduced CCFN (6 files) had 68% less respiration, 15% less optimum nitrogen content, 36% less minimum nitrogen content, and 57% smaller root diameter (Table 1) compared with increased CCFN (18 files). Large CCS (450 µm^2^) had 73% less respiration, 9% less optimum nitrogen content, 10% less minimum nitrogen content, and 22% larger root diameter (Table 1) compared with small CCS (200 µm^2^). It is noteworthy that reduced CCFN and large CCS have the smallest tissue construction and maintenance cost in terms nitrogen content and respiration, but with the difference that large CCS increases root diameter (Supplemental Figs. S2 and S3), representing a potential increase in root maintenance cost at whole root system scale. We built 4 independent models to predict respiration, optimum nutrient content, minimum nutrient content, and root diameter utilizing the phenotypic variation of CCFN and CCS as predictors.

**Table 1.** Multiple linear regression predictions of respiration, root diameter, and optimum and minimum nitrogen content for the following phene states of CCFN and CCS: reduced CCFN, increased CCFN, large CCS, and small CCS

Phene state	CCFN	CCS (µm^2^)	Respiration (g CO_2_ g^−1^ DW d^−1^)	Optimum_N (µmol g^−1^)	Minimum_N (µmol g^−1^)	Diameter (mm)
Reduced CCFN	6	360	0.014	966.1	515.38	0.69
Increased CCFN	18	360	0.044	1,141.02	816.66	1.62
Large CCS	10	450	0.012	989.49	591.46	1.07
Small CCS	10	200	0.046	1,086.48	659.09	0.88

### Transphenic analysis of CCFN and CCS components

**Figure 2.**
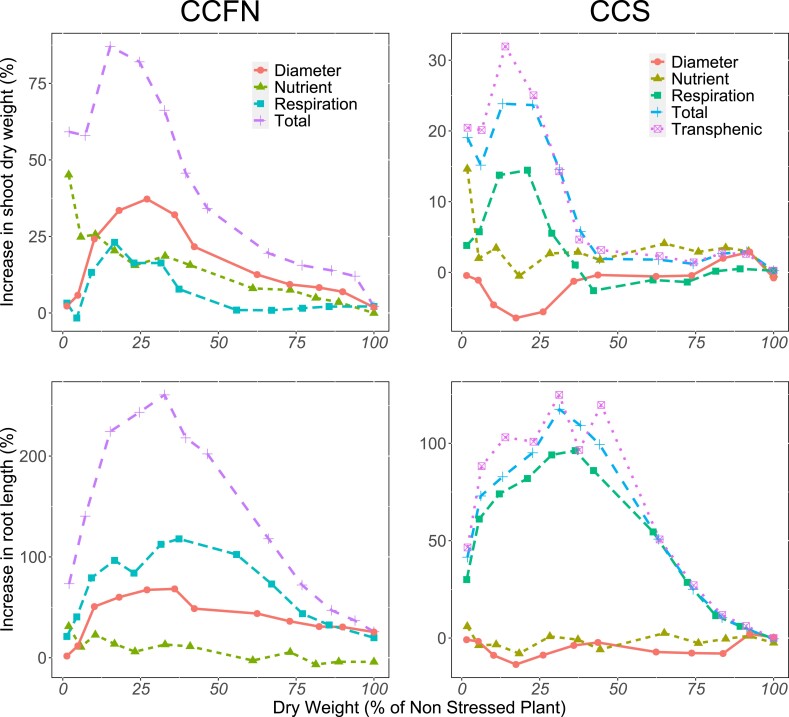
Relative benefit of respiration, nitrogen content, and root diameter of reduced CCFN and large CCS phenotypes. The *x* axis shows the performance of the simulated plants under varying levels of nitrogen stress, where 100% is the absence of stress. The *y* axis shows the benefit in shoot dry weight and root length relative to the reference phenotypes (increased CCFN and small CCS, respectively). “Total” represents the combined effect of respiration, nutrient content, and root diameter. “Transphenic” represents the large CCS phenotype using the root diameter of small CCS.

Reduced CCFN and large CCS showed differential reduction of respiration and nutrient content. Large CCS increased root diameter, in contrast with the decrease in root diameter by reduced CCFN (Supplemental Fig. S3). To evaluate the independent contributions of root respiration, nitrogen content, and root diameter to plant performance, we used a transphenic analysis by simulating the effect of each variable independently. When the components of reduced CCFN were simulated independently, we observed a 23%, 20%, and 33% increase in shoot dry weight compared with increased CCFN for respiration, nitrogen content, and root diameter, respectively (Fig. 2). The additive effects of these components correspond to a 73% increase in shoot dry weight; however, when the components were simulated together (Total), we observed an 82% increase in shoot dry weight, showing a synergistic effect (Fig. 2). We also observed a contribution of 97%, 14%, and 60% in the increase of root length for respiration, nitrogen content, and root diameter, respectively. The expected additive effect was 171%; however, when the components were simulated together (Total), we observed an increase in root length of 243% compared with the increased CCFN phenotype, showing a synergistic effect for increased root length (Fig. 2). For CCFN, the root diameter had the greatest benefit in shoot dry weight accumulation and root length, followed by respiration and nutrient content (Fig. 2). When we simulated the components of large CCS independently, respiration and nutrient content increased the shoot dry weight by 13% and 3%, respectively (Fig. 2). However, root diameter decreased the shoot dry weight by 4% compared with the small CCS phenotype. The expected additive increase in shoot dry weight was 12%; however, when we simulated the components together, we observed a 24% increase in shoot dry weight, showing that the benefits of respiration and nutrient content work in synergy and offset the effect of large root diameter. Furthermore, when we transferred the root diameter of small CCS to large CCS to create a new category called “transphenic,” we observed a total benefit of 32% with respect to small CCS, showing that the increase in root diameter lessened the potential benefits of large CCS (Fig. 2). For root length, we observed an independent contribution of 94% for respiration, 1% for nitrogen content and −9% for root diameter, giving a total additive effect of 86%. However, when we simulated the components together, we observed an increase in root length of 117%, showing a synergistic effect. For the transphenic phenotype, we observed an increase in root length of 125%, showing that decreasing the root diameter would improve plant performance. For CCS, respiration was the main factor contributing to the increase in shoot dry weight, followed by nitrogen content. In summary, the synergy or antagonistic effect of root diameter on shoot dry weight or root length is a function of the phene states of CCFN and CCS. While root respiration and nutrient content increased shoot dry weight under low-nitrogen stress for reduced CCFN and large CCS, reduced CCFN showed a higher increment compared with large CCS.

### Sensitivity analysis

**Figure 3.**
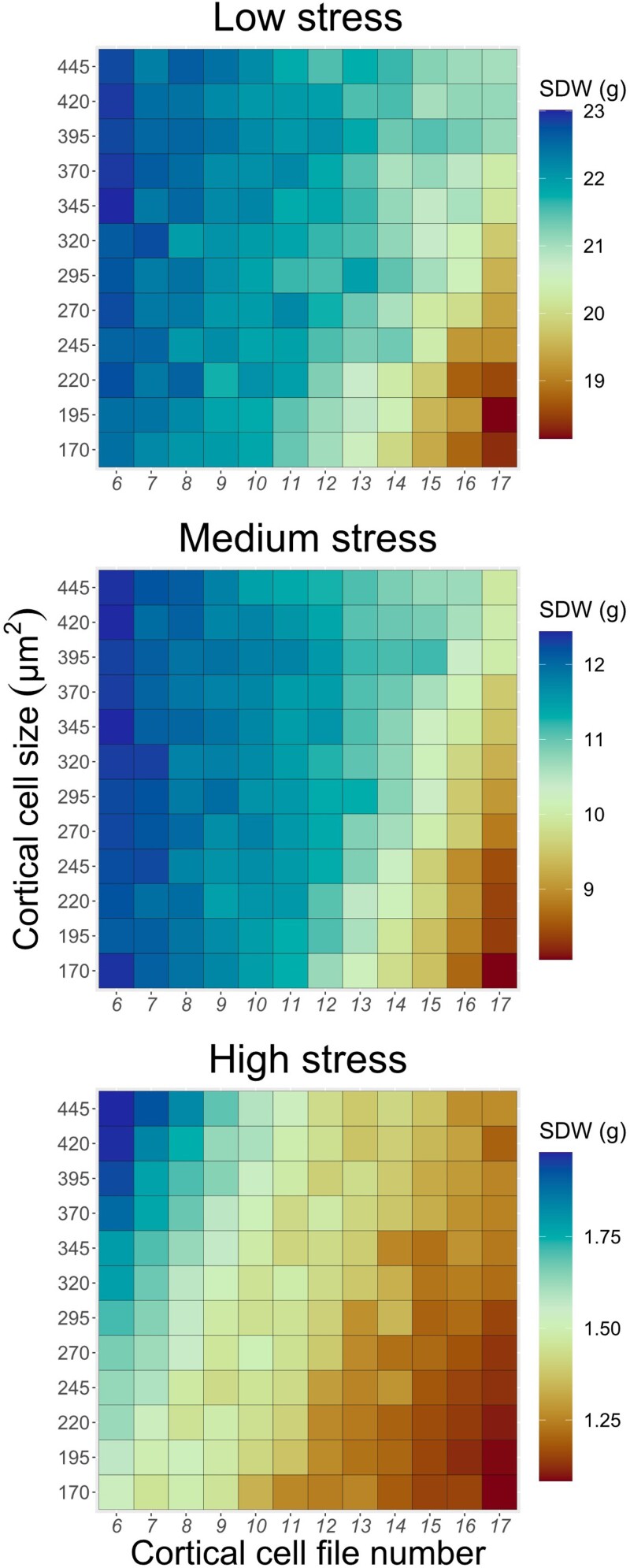
Sensitivity analysis of phenotypic variation for cortical cell file number and cortical cell size under low-, medium-, and high-nitrogen stress and the effect on shoot dry weight (SDW). Low, medium, and high stress corresponds to an average of 25%, 50%, and 75% shoot biomass reduction compared with a nonstressed plant, respectively.

To evaluate the effect of multiple combinations of CCFN and CCS under different levels of nitrogen stress, we simulated varying CCFN and CCS under 3 levels of nitrogen availability (low stress, medium stress, and high stress). Under high stress, values >370 mm^2^ for CCS and <9 for CCFN were necessary to produce a shoot dry weight >1.75 g (Fig. 3). Under medium- and low-nitrogen stress, 12 or fewer CCFN was enough to produce a shoot dry weight value above 11 and 21 g, respectively (Fig. 3). When CCFN was <10 files, the effect of having large CCS on shoot dry weight was very small under low and medium stress. However, when the plants experienced high-nitrogen stress, the benefit of large CCS was greater than small CCS at any value of CCFN (Fig. 3). The more nitrogen stress, the plant experiences the more extreme phene state is needed to improve plant growth.

### Interaction of CCFN/CCS with RCA

**Figure 4.**
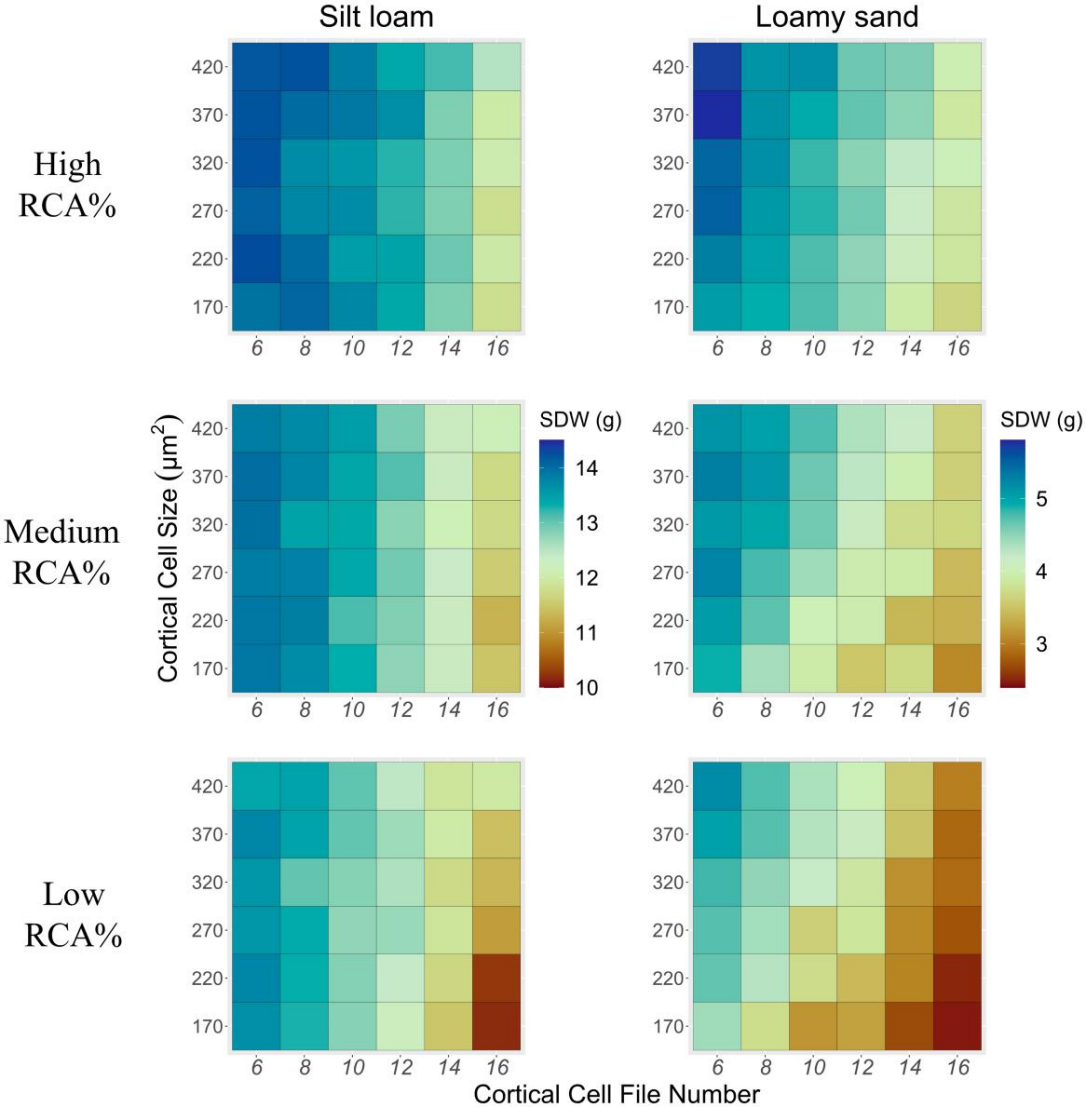
Effect of the interaction between CCFN, CCS, and RCA on shoot dry weight (SDW) under suboptimal nitrogen availability in silt loam and loamy sand soils. The scale bar shows different values for shoot dry weight. Aerenchyma levels correspond to 10%, 20%, and 30% for low, medium, and high, respectively).

Different combinations of phene states create multiple integrated phenotypes that could be beneficial or detrimental under specific environments (Dathe et al. 2016; Rangarajan et al. 2018; Klein et al. 2020). To test the interaction of phene states of CCFN and CCS with RCA, we performed simulations for the phenotypic distribution of CCFN, CCS, and RCA under low nitrogen in silt loam and loamy sand soils. We observed a greater reduction in shoot dry weight in silt loam compared with loamy sand due to the greater nitrogen leaching in loamy sand (Fig. 4). Reduced CCFN showed a greater shoot growth in silt loam, while large CCS had greater shoot growth in loamy sand. Greater RCA improved shoot dry weight in both environments. In loamy sand, the increase in shoot dry weight due to RCA was homogeneous along different levels of CCFN and CCS; however, in silt loam, the increase in shoot dry weight due to RCA was greater in combination with increased CCFN and small CCS (Fig. 4). In summary, in loamy sand, all combinations for CCFN and CCS improve plant growth with high levels of RCA, while in silt loam, the reduced CCFN and large CCS showed less benefit from high RCA compared with increased CCFN and small CCS.

### Interaction of CCFN/CCS with LRBD

**Figure 5.**
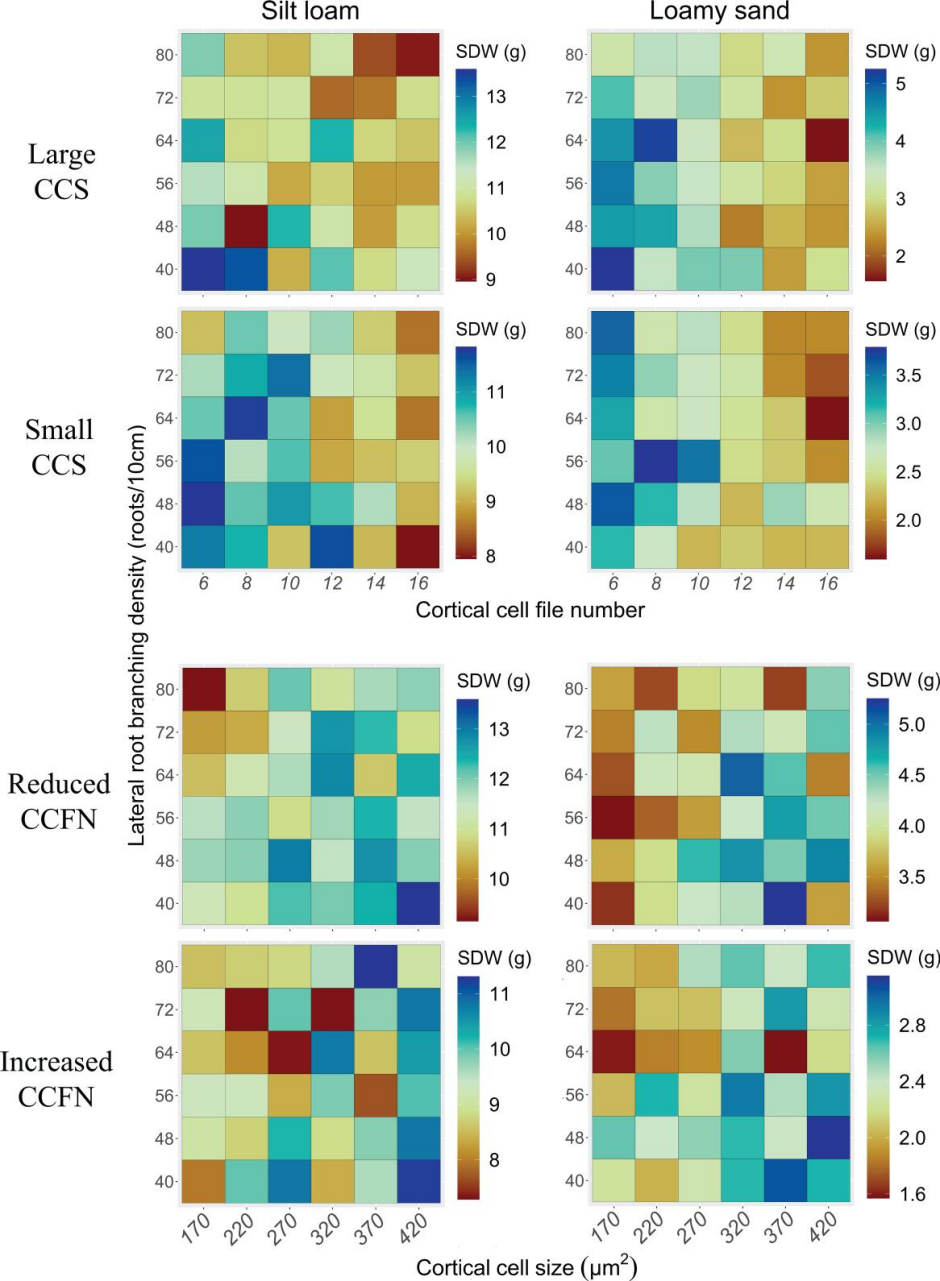
Effect of the interaction between LRBD, CCFN, and CCS on shoot dry weight (SDW) under suboptimal nitrogen availability in silt loam and loamy soils.

To test the interaction between CCFN and CCS with LRBD, we performed simulations corresponding to a representative set of combinations of the distribution for these phenes under nitrogen stress in silt loam and loamy sand soils. We observed that several LRBD phenotypes increased plant growth when combined with other specific phene states and soil textures. In loamy sand, high LRBD increased shoot dry weight when the root metabolic cost was small, for instance, when combined with reduced CCFN/large CCS. However, when the metabolic cost was high, for instance, combined with increased CCFN/small CCS, high LRBD was detrimental to plant growth (Fig. 5 and Table 2). In silt loam, low LRBD improved plant growth when the root metabolic cost was small (reduced CCFN/large CCS), but it became detrimental when the metabolic cost was high (increased CCFN/small CCS; Table 2). In summary, the relative improvement or detrimental effects in shoot growth due to high or low LRBD depend on the soil texture and the root metabolic cost given by the interaction with other phenes such as CCFN and CCS.

**Table 2.** Relative increase in shoot dry weight of multiple integrated phenotypes influenced by variation in CCFN, CCS, RCA, and LRBD

Integrated root phenotypes	Silt loam	Loamy sand
	Increase in shoot dry weight^a^ (%)	Increase in shoot dry weight^a^ (%)
RCA		
ReducedCCFN/LargeCCS/HighRCA	43.8	145.1
ReducedCCFN/SmallCCS/HighRCA	40.9	114.4
ReducedCCFN/LargeCCS/LowRCA	39.5	114.8
ReducedCCFN/SmallCCS/LowRCA	37.4	71.1
IncreasedCCFN/LargeCCS/HighRCA	26.5	74.0
IncreasedCCFN/SmallCCS/HighRCA	21.5	59.6
IncreasedCCFN/LargeCCS/LowRCA	20.7	38.0
IncreasedCCFN/SmallCCS/LowRCA	0.0	0.0
LRBD		
ReducedCCFN/LargeCCS/LowLRBD	86.4	128.7
ReducedCCFN/LargeCCS/HighLRBD	63.4	182.3
ReducedCCFN/SmallCCS/LowLRBD	54.8	101.4
IncreasedCCFN/LargeCCS/LowLRBD	54.0	72.6
ReducedCCFN/SmallCCS/HighLRBD	25.6	128.9
IncreasedCCFN/LargeCCS/HighLRBD	24.1	70.8
IncreasedCCFN/SmallCCS/HighLRBD	17.2	29.3
IncreasedCCFN/SmallCCS/LowLRBD	9.1	42.5

These results are outputs from *OpenSimRoot.*

Increase percentage in shoot dry weight is relative to the worst performer in each category.

## Discussion

OSR is a functional-structure model of plant–soil interactions designed to analyze the fitness landscape of root phenotypes. Here, we used OSR to test the hypothesis that reduced CCFN and large CCS, which improve the growth and yield of maize under water deficit (Chimungu et al. 2014a, 2014b), would also be beneficial in soils with suboptimal nitrogen availability. In support of this hypothesis, both reduced CCFN and large CCS were associated with substantial improvements in nitrogen capture and plant growth under nitrogen stress. Under nitrogen stress, reduced CCFN improved shoot growth up to 82% and large CCS improved shoot growth up to 24% compared with increased CCFN and small CCS (Table 1 and Fig. 2). Large root diameter showed a negative impact on shoot dry weight in phenotypes with large CCS. Phenotypes with large CCS yet a small root diameter had 32% better growth than phenotypes with small CCS under nitrogen stress (Fig. 2). A combination of reduced CCFN and large CCS showed the maximum benefit in low-, medium-, and high-nitrogen stress. Integrated phenotypes with reduced CCFN, large CCS, and high RCA increased shoot biomass under nitrogen stress by 44% in a silt loam and 145% in a loamy sand (Table 2 and Fig. 4). Finally, integrated phenotypes with reduced CCFN, large CCS, and low LRBD were the best performers in a silt loam, but phenotypes with high LRBD were the best performers in a loamy sand (Table 2 and Fig. 5). Our results align with previous reports that RCA formation, which reduces the metabolic cost of soil exploration in terms of nutrient and C investment, improves plant growth under conditions of suboptimal availability of N (Postma and Lynch 2011b; Saengwilai et al. 2014a; Galindo-Castañeda et al. 2019), and supports the hypothesis that larger CCS and reduced CCFN increase soil nutrient acquisition by reducing root metabolic costs.

### Reduced CCFN and high RCA: an integrated phenotype for compacted soils

It is noteworthy that reduced CCFN improved plant growth more than large CCS. We observed that reduced root diameter was primarily responsible for the utility of reduced CCFN under nitrogen stress, while in contrast, large CCS increased root diameter. It has been proposed that root thickening would improve soil exploration of compacted soils by expanding the soil and filling it axially (Atwell 1993). However, Vanhees et al. (2020) found no evidence that larger root diameter improved rooting depth in compacted soils, instead, they found that reduced CCFN and large CCS increased rooting depth under compacted soils. On the other hand, Colombi et al. (2019) showed that in wheat (*Triticum aestivum*), the energy cost of root growth in compacted soils increases by up to 90% and that a reduction in the energy cost of root growth due to large cortical cell diameter is a useful adaptation for compacted soils. Although large CCS decreases the cell surface–volume and cytoplasm–vacuole ratios which reduces metabolic cost, larger CCS also increases radial volume, which increases respiration. On the other hand, phenotypes with reduced CCFN have fewer respiring cells and less volume; therefore, the total reduction of metabolic cost is greater compared with large CCS. Aerenchyma was also associated with greater rooting depth under compaction (Colombi and Walter 2016; Vanhees et al. 2020) and may have other advantages for subsoil exploration such as greater oxygenation (Lynch and Wojciechowski 2015). Therefore, we propose that an integrated phenotype composed of reduced CCFN and high RCA will improve growth under low nitrogen but also would be useful for compacted soils.

### Interaction of RCA with CCFN and CCS

In most high-input agroecosystems, the predominant form of nitrogen is nitrate which is soluble in water and leaches to the subsoil with precipitation (Lynch and Wojciechowski 2015). It has been demonstrated that root traits that promote greater root depth increase the capture of nitrate that leaches and improve the plant growth under nitrogen stress (Saengwilai et al. 2014b). For instance, root phenotypes with fewer nodal roots showed a reduced metabolic cost of root maintenance and deeper rooting depth compared with maize varieties with a high number of crown roots (Saengwilai et al. 2014b). Root anatomical traits such as RCA have proved to improve plant growth under low-nitrogen conditions due to the decrease in root metabolic cost and increased rooting depth (Saengwilai et al. 2014a). It is important to consider that the effect of root traits on the nitrogen capture will interact with the environmental and management factors, especially those which affect the nitrogen distribution and leaching (Lynch and Wojciechowski 2015). Our findings showed that in a loamy sand, the best-performing phene combination was reduced CCFN, large CCS, and high RCA. For silt loam soils, the contribution of RCA to plant growth was smaller when combined with reduced CCFN and large CCS; however, the greatest benefit of RCA was observed in combination with increased CCFN and small CCS. The higher improvement in plant growth by RCA in loamy sand soil might be related to the leaching of nitrogen. Since in silt loam, soil nitrogen leaches less, there is more nitrogen available and the reduction in metabolic cost given by reduced CCFN and large CCS is enough to improve growth. However, in loamy sand soils, the high leaching produces higher stress, and the reduction of metabolic cost given solely by reduced CCFN and large CCS is not enough to produce the maximum potential improvement in growth, so adding high RCA would improve plant performance to its maximum potential. Our findings showed that combining RCA with reduced CCFN and large CCS improved plant growth under sandy loam soils; however, the effect of adding aerenchyma was minor under silt loam soils.

### Interaction of LRBD with CCFN and CCS

It has been demonstrated that maize lines with many and short lateral roots have better topsoil exploration, improving plant performance under low phosphorus stress (Postma et al. 2014; Jia et al. 2018). In contrast, maize lines with few and long lateral roots have improved rooting depth in the subsoil and increased plant growth in low nitrogen (Zhan and Lynch 2015) and under water deficit (Zhan et al. 2015). The optimal lateral root density depends on nutrient mobility (Postma et al. 2014). For instance, an in silico study showed that >9 lateral roots per centimeter of axial root would be optimal for the capture of immobile nutrients such as phosphorus; however, <7 would produce longer roots that decrease competition for resources and increase the capture of mobile nutrients like nitrate (Postma et al. 2014). Our findings showed that in loamy sand with high rates of nitrogen leaching, high LRBD improves plant growth when combined with other phene states that reduce the root metabolic cost, for instance, reduced CCFN and large CCS. The greater root maintenance cost associated with greater lateral branching density is compensated by a decrease in root respiration from reduced CCFN and large CCS. On the other hand, in phenotypes with increased CCFN and small CCS, having low LRBD represents a better adaptation in a loamy sand, which agrees with Postma et al. (2014). In a silt loam, low LRBD combined with reduced CCFN and large CCS was the best-performing integrated phenotype because there is less mobility of nitrate. When nitrogen mobility is reduced due to the silt loam soil, smaller LRBD would reduce root competition for nitrogen and increase the uptake per unit of root length (Postma et al. 2014). Our findings are consistent with the observations that reduced LRBD improved growth and yield in maize under drought (Zhan et al. 2015) and nitrogen (Zhan and Lynch 2015) stress, and that high LRBD improves plant performance under stress for nonmobile nutrients (Postma et al. 2014; Jia et al. 2018). Furthermore, our results highlight the importance of phene interactions and the soil environment in structuring the fitness landscape of root phenotypes.

### Model considerations

OSR has several assumptions and limitations that should be considered. In this study, we used *OpenSimRoot* version 1 (Postma et al. 2017), so the effects of drought and new shoot capabilities from *OpenSimRoot_v2* (Schäfer et al. 2022a) are not included. OSR does not simulate phytohormones and explicit transport and assimilation of nutrients through the plant. Due to the increase in complexity and the computational demand of root interactions, the root system development is simulated for up to 42 d. Root growth is not affected by temperature or O_2_ availability. Microbial interactions with roots and soil are not simulated. Yet, the available modules that simulate root carbon cost, nutrient dynamics, and stress responses are representative of the phenomena, we are studying, as shown by good agreement between *SimRoot* results (Postma and Lynch 2011b, 2012; Postma et al. 2014) and field results (Saengwilai et al. 2014a, 2014b; Zhang et al. 2014; Zhan and Lynch 2015). An important merit of simulation modeling is the ability to test hypotheses and probe scenarios that are inaccessible to empirical studies. Simulation modeling makes it possible to isolate and test the objects of study; in this case, larger CCS and reduced CCFN, from interactions with many other biotic and abiotic factors, which is difficult to avoid in empirical studies (Dunbabin et al. 2013; Postma et al. 2014; Lynch et al. 2021). Modeling also permits the analysis of phenotypes that may not exist or would be challenging to generate empirically, such as changing nutrient uptake kinetics (York et al. 2016), or examining root competition in time and space in the “3 sisters” polyculture (Postma et al. 2014), where empirical measurement is impractical. Because of its heuristic nature, OSR focuses on the validity of simulating physiological processes, rather than the alignment with empirical studies that predictive models emphasize. Heuristic models like OSR are useful complements to empirical research because they rigorously and quantitively test the adequacy of the logic model that supports our interpretation of the phenomena being modeled.

## Conclusions

Quantitative evidence is presented that larger CCS and reduced CCFN are adaptive phene states for nitrogen capture under suboptimal nitrogen availability. The utilities of larger CCS and reduced CCFN in soils with suboptimal nitrogen availability are affected by nutrient availability, soil type, and interactions among phenes. We propose that larger CCS and reduced CCFN are complementary to RCA formation in terms of growth enhancements, especially in sandy loam soils and with minor effect on silt loam soils. Interactions between LRBD, CCS, and CCFN improve growth under nitrogen stress when the phene states are synchronized with respect to the soil texture. We expect tradeoffs for RCA formation, larger CCS, and reduced CCFN to be present, as all 3 phene states may be related to the penetration of hard soil domains. This aspect merits further investigation. Functional-structural plant models like OSR can be used to simulate root anatomical phenotypes and thereby evaluate their utilities under multiple edaphic stresses and have the potential to provide a holistic understanding of the roles of root phenotypes for plant fitness. These results indicate that large CCS and reduced CCFN merit investigation as breeding targets for maize and possibly other cereal crops, since the development of crop cultivars with improved soil resource acquisition remains a critical strategy for improving the sustainability of intensive agriculture and for improving the productivity of low-input agroecosystems.

## Materials and methods

We used OSR (Postma et al. 2017), a functional-structural plant model with focus on root architecture and soil resource acquisition, to simulate the formation of RCA, variation in LRBD, CCS, and CCFN, and their physiological utility in maize (*Z. mays*) growing with varied nitrate availability in the soil. We also evaluated potential additive effects among RCA, LRBD, CCS, and CCFN.

### Model description

OSR is a dynamic, spatially explicit model of soil resource capture by root systems (Ajmera et al. 2022; Rangarajan et al. 2022; Schäfer et al. 2022a). The root system is described as distinct root classes represented by a growing number of root nodes and segments as the root system develops (Lynch et al. 1997). Root growth is based on a carbon source-sink model, where the carbon partition protocol has been described by Postma and Lynch (2011a). Shoot growth and photosynthesis are simulated using LINTUL (Light INTerception and UtiLization simulator) model (Spitters and Schapendonk 1990). Nutrient uptake is simulated for each root segment in comparison with the optimal and minimal nutrient requirements of the plant. Nutrient deficiency, or stress factor, is defined as when nutrient uptake falls below the optimal nutrient requirement. The stress factor influences shoot development and photosynthetic efficiency depending on the nutrient simulated. OSR is linked to the 3D hydrological model SWMS3D (Simunek et al. 1995) to simulate nitrate uptake.

Variation in RCA formation, LRBD, CCS, and CCFN in maize are simulated for each root segment with empirical parameters retrieved from Saengwilai et al. (2014a), Jia et al. (2018), and Chimungu et al. (2014a, 2014b), respectively. The percentage RCA for different root classes is described by Fan et al. (2003) and Saengwilai et al. (2014a). We simplified CCS and CCFN simulation by assuming they are uniformly distributed across root classes. The addition of CCS and CCFN were implemented as new model input files, no specific modifications were made to the computational codes of OSR to accommodate this addition. RCA formation combines nutrient remobilization and respiration reduction and is based on regression between the amount of RCA and nutrient content and root respiration of empirical measurements by Fan et al. (2003). OSR does not explicitly represent root anatomy; so CCS and CCFN variations are represented by reducing modeled root respiration and tissue nutrient content.

### Effects of nutrient stress on growth

In OSR, the nutrient stress factor module is implemented to affect the potential leaf area expansion rate and light use efficiency (LUE) independently as in the LINTUL model. The nutrient stress factor functions as a growth regulator between root and shoot growth. The nutrient stress factor negatively impacts LUE and resulted in reduced carbon available for plant growth. Reduction in the potential leaf area expansion rate caused by the stress factor results in reduced sink strength of the shoot, and consequently greater allocation of carbon to root growth. Nutrient-specific stress responses were used to determine the effect of internal nutrient concentrations (nitrogen) on the 2 parameters. Inorganic nitrogen strongly affects both parameters (Uhart and Andrade 1995).

### Distribution of RCA formation, CCS, and CCFN within the root system

We assumed that RCA formation starts behind the elongation zone of a root and develops over time until reaching a maximum. Therefore, the greatest amount of RCA formation can be found close to the base of a root, which aligns with Fan et al. (2003) but disagrees with Bouranis et al. (2003), and Burton et al. (2012). RCA formation is reduced in the first 5 cm of the root (Bouranis et al. 2003), which is a small part of the total root length, and we expect the effect on total RCA formation to be small. We used the maximum amount of RCA formation in the literature, which is 39% of root cross-section area at 20 days after germination (Fan et al. 2003) in the model.

Variation in CCS and CCFN was observed in the mid cortical band of roots by Chimungu et al. (2014a, 2014b). In reality, the spatial distribution of CCS and CCFN is not uniform in either the area cross-sectioned or the across different root classes. To demonstrate the effect of observed respiration reduction of these phenes, we assumed that CCS and CCFN variations are uniform regardless of root class and location in the area cross-sectioned. Parameterization of these phenes is based on the genotypic variation described by Chimungu et al. (2014a, 2014b). CCS varies between 170 and 445 μm^2^, and CCFN varies between 6 and 18 in maize.

### Interactions between RCA formation, LRBD, CCS, and CCFN

Living cortical area (LCA; Jaramillo et al. 2013) is proposed as a good predictor of root respiration, and a critical determinant of root metabolic cost, which involves the phenes in this study. Interactions between LCA components require further demonstration. We simulated the extremes of variation in RCA formation, where RCA takes up between 0% and 30% of the root cross-sectional area, CCS, and CCFN to probe additive effects under low nitrogen. Substantial genetic variation exists in LRBD (Zhan et al. 2015). We varied the LRBD parameter to the extremes reported in this study, between 4 and 8 lateral roots/cm on axial roots, to examine if potential synergism between LRBD, CCS, and CCFN under low soil nitrogen to further test the utility of CCS and CCFN in an integrated genotype.

### Parameter collection

We selected 3 genotypes for each of the following categories previously reported by Chimungu et al. (2014a, 2014b): large CCS, small CCS, increased CCFN, and decreased CCFN (Supplemental Table S1). Three plants for each genotype were planted in 10 L pots with 10% soil, 50% sand, 20% vermiculite, and 20% perlite. The soil (Typic Hapludalf, silt loam) was collected from low-nitrogen fields which have not receive nitrogen fertilizer over a decade at the Russell E. Larson Agricultural Research Center in Rock Springs, Pennsylvania (77°57′W, 40°42′N). Nutrient solution was provided daily for 2 min. For the high- and low-nitrogen solutions, we used 1,500 and 260 µmol of NH_4_NO_3_, respectively. The concentration of the rest of the nutrients was: MgSO_4_ 1000 µmol, K_2_SO_4_ 500 µmol, Ca(PO_4_) 250 µmol, Fe-DTPA 150 µmol, CaSO_4_ 1,500 µmol, H_3_BO_3_ 46 µmol, MnCl_2_ 1.81 µmol, ZnSO_4_ 0.73 µmol, CuSO_4_ µmol, and (NH_4_)_6_Mo_7_O_24_ 0.074 µmol. Plants were grown in September 2021, at Pennsylvania State University (40°48′ N, 77°51′W) under controlled conditions (14 h of day at 28°C/10 h of night at 24°C, 40% to 70% relative humidity). Plants were harvested 30 after planting. Roots were washed and second nodal roots were sampled 5–10 cm from the base for respiration, anatomy, and nitrogen content determination. Respiration was measured with a LI-6400 Portable Photosynthesis System (Li-COR Biosciences), utilizing a custom chamber (3.2973 cm^3^) attached to the closed-system IRGA. The temperature was constant at 24°C ± 1.9, and the flow rate was 400 µmol of CO_2_. CO_2_ evolution was recorded every 5 s for 180 s. For anatomy, second nodal roots were segmented and stored in 75% ethanol in water and then were processed utilizing laser ablation tomography (Strock et al. 2019). To quantify the anatomical root phenotypes, we utilized RootScan 2.3v (Burton et al. 2012). Tissue nitrogen content was determined utilizing 2400 CHNS/O Series II element analyzer (PerkinElmer).

### System description, parameterization, and runs

We simulated growth of 40 days after germination of a single maize plant, which represents a uniform monoculture plant community with a between-row spacing of 60 cm and a within row spacing of 26 cm. Aboveground competition was simulated by a shading function (Postma and Lynch 2011a). Parameterization was based on input parameters used in the previous simulation studies with OSR (Postma and Lynch 2011a, 2011b). To parameterize the model, we measured respiration, nitrogen content, and root diameter in 12 maize genotypes previously categorized as reduced CCFN (3), increased CCFN (3), small CCS (3), and large CCS (3; Supplemental Table S1). We observed phenotypic variation for CCS, CCFN, respiration, nitrogen content, and root diameter under high- and low-nitrogen conditions (Supplemental Fig. S1 and Fig. S3). We used 4 independent multiple linear regression models to predict respiration, optimum nutrient content, minimum nutrient content, and root diameter utilizing CCFN and CCS values as predictors (Supplemental Fig. S2). We predicted respiration, optimum nutrient content, minimum nutrient content, and root diameter for the whole variation a range between 6 and 17 CCFN and 170 and 440 μm^2^ and used those predictions as inputs for the simulations in OSR. We used the RCA function in OSR to combine the variation of CCFN and CCS with values of RCA that ranged from 5% to 30%. For the interactions with LRBD, we used values that ranged from 4 to 8 roots per cm. All simulations were performed on the Penn State supercomputing clusters. To account for stochasticity in growth rates and root branching frequencies, each scenario was simulated with 4 replications each with OSR's random number generator initialized to different values, with the graph showing the mean value. The variation of phenes in this study was based on empirical studies to avoid extrapolation toward unrealistic conditions. We used ParaView 5.9.0 (Henderson 2015) analyze the 3D root system architecture. The repository located here: https://github.com/ilovaldivia/CCFN-CCS-RCA-LRBD contains all the input files utilized in this study.

### Statistical analysis

Multiple linear regression models to predict respiration, optimum nutrient content, minimum nutrient content, and root diameter were implemented utilizing R 4.2.1 (R Core Team 2019). Output root systems were visualized utilizing ParaView 5.9.1-RC3 (Kitware Inc). We did not conduct significance tests for the simulation results, as such tests are not reliable in simulation studies, as the ease of replication in computer simulations allows for any effect size to be found significant if there are enough replicates. Biological importance, rather than the statistical significance, should be the main focus of simulation experiments (White et al. 2014).

## Supplementary Material

kiad214_Supplementary_Data

## Supplemental data

The following materials are available in the online version of this article.

**Supplemental Figure S1.** Phenotypic variation of root anatomy and physiological parameters measured in the greenhouse for 12 maize inbred lines previously reported by Chimungu et al. (2014a, 2014b).

**Supplemental Figure S2.** Multiple linear regression models to estimate respiration, optimum nitrogen, minimum nitrogen, and root diameter utilizing the phenotypic variation of cortical cell file number (CF_num) and cortical cell size as predictors.

**Supplemental Figure S3.** Independent correlations between cortical cell file number and cortical cell size and root respiration, minimum and maximum nitrogen content, and root diameter.

**Supplemental Table S1.** Genotypes for reduced CCFN, increased CCFN, large CCS, and small CCS from Chimungu et al. (2014a, 2014b).
