# Supplementary material for: Large root cortical cells and reduced cortical cell files improve growth under suboptimal nitrogen in silico
Source: Plant Physiol. 2023 Apr 11;192(3):2261–75. doi: 10.1093/plphys/kiad214 (PMC10315315; doi:10.1093/plphys/kiad214)
Supplement: kiad214_Supplementary_Data [file kiad214_supplementary_data.pdf]

Supplemental Data

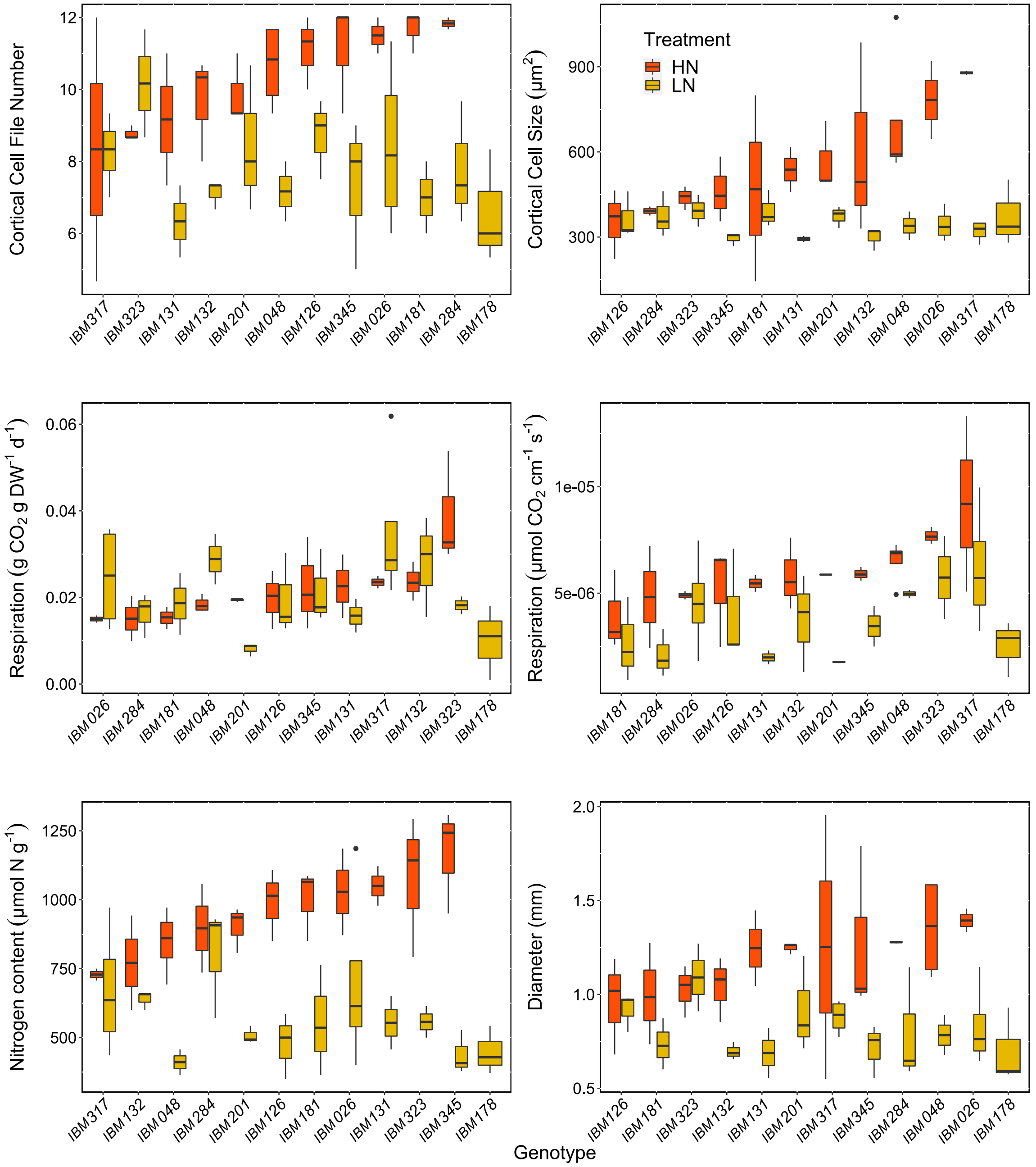

Supplemental Figure S1: Phenotypic variation for root anatomical and physiological parameters measured in the greenhouse for 12 maize inbred lines previously reported by Chimungu (2014a) and Chimungu (2014b). Treatments correspond to high nitrogen (HN) and low nitrogen (LN). Centerline = median; box limits = upper and lower quartiles; whiskers = 1.5x interquartile range; points = outliers.

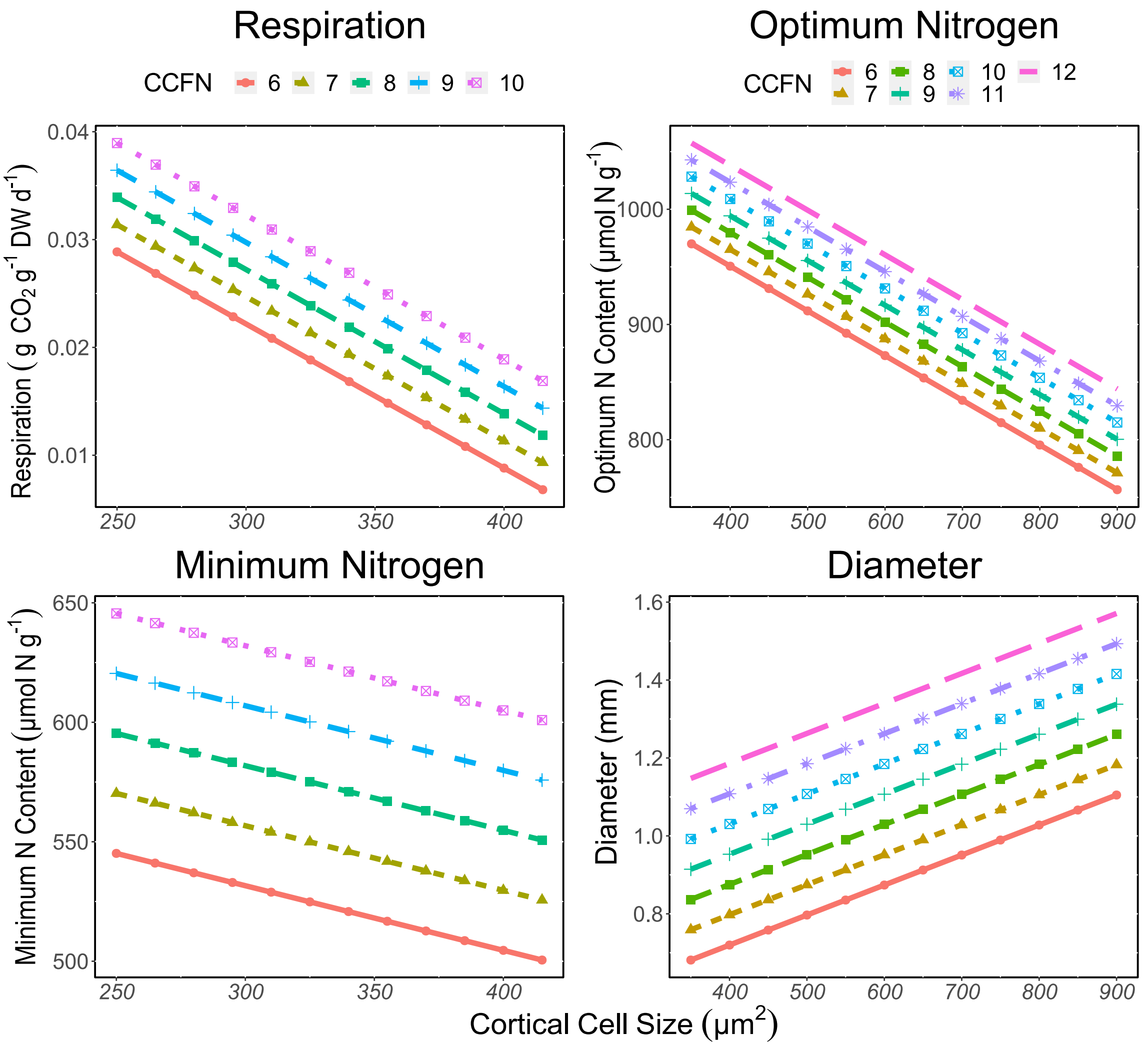

Supplemental Figure S2: Multiple linear regression models to estimate respiration, optimum nitrogen, minimum nitrogen, and root diameter utilizing the phenotypic variation for cortical cell file number (CCFN) and cortical cell size as predictors.

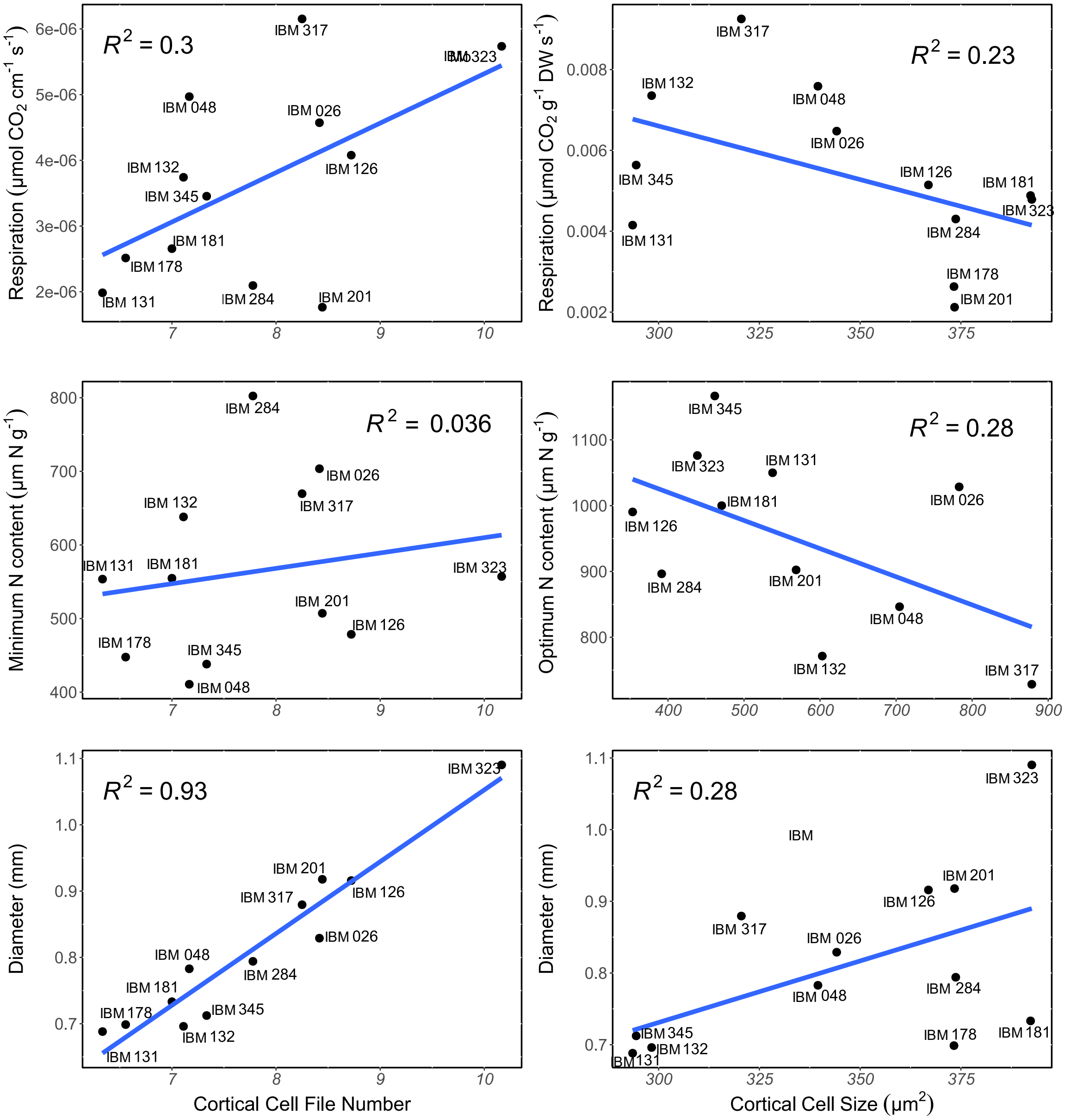

Supplemental Figure S3: Correlations between cortical cell file number and cortical cell size and root respiration, minimum and maximum nitrogen content, and root diameter.

Supplemental Table S1. Genotypes contrasting for CCFN and CCS from Chimungu (2014a,b).

Phenotype	Phene state	Genotypes
CCS	Large	IBM026, IBM126, IBM201
	Small	IBM131, IBM132, IBM323
CCFN	Increased	IBM181, IBM284, IBM345
	Reduced	IBM048, IBM317, IBM178
